# Dystonia with Brain Manganese Accumulation Resulting From *SLC30A10* Mutations: A New Treatable Disorder

**DOI:** 10.1002/mds.25138

**Published:** 2012-08-23

**Authors:** Maria Stamelou, Karin Tuschl, W K Chong, Andrew K Burroughs, Philippa B Mills, Kailash P Bhatia, Peter T Clayton

**Affiliations:** 1 Sobell Department of Motor Neuroscience and Movement Disorders, University College London Institute of NeurologyLondon, United Kingdom; 2 Clinical and Molecular Genetics Unit, University College London Institute of Child HealthLondon, United Kingdom; 3 Department of Radiology, Great Ormond Street Hospital for ChildrenLondon, United Kingdom; 4 The Liver, Pancreatic, Biliary and Transplant Unit, The Wellington HospitalLondon, United Kingdom

**Keywords:** dystonia, hypermanganesemia, cirrhosis, polycythemia, SLC30A10

## Abstract

**Background:**

The first gene causing early-onset generalized dystonia with brain manganese accumulation has recently been identified. Mutations in the *SLC30A10* gene, encoding a manganese transporter, cause a syndrome of hepatic cirrhosis, dystonia, polycythemia, and hypermanganesemia.

**Methods:**

We present 10-year longitudinal clinical features, MRI data, and treatment response to chelation therapy of the originally described patient with a proven homozygous mutation in *SLC30A10*.

**Results:**

The patient presented with early-onset generalized dystonia and mild hyperbilirubinemia accompanied by elevated whole-blood manganese levels. T1-sequences in MRI showed hyperintensities in the basal ganglia and cerebellum, characteristic of manganese deposition. Treatment with intravenous disodium calcium edetate led to clinical improvement and reduction of hyperintensities in brain imaging.

**Conclusions:**

We wish to highlight this rare disorder, which, together with Wilson's disease, is the only potentially treatable inherited metal storage disorder to date, that otherwise can be fatal as a result of complications of cirrhosis. © 2012 Movement Disorder Society

The first inborn error of manganese metabolism has recently been identified.^1^ This autosomal recessive condition caused by mutations in the *SLC30A10* (Solute Carrier Family 30, Member 10) gene, encoding a manganese transporter, results in manganese accumulation, mainly in the basal ganglia and cerebellum, and the liver, and causes a syndrome of early-onset generalized dystonia, cirrhosis, polycythemia, and hypermanganesemia.^1^–^3^

Twenty affected individuals from 10 families have been described by two independent groups.^1^, ^3^ Seventeen affected members from eight families presented with young-onset (2–14 years) generalized dystonia, whereas 1 affected member presented with spastic paraparesis without dystonia. Interestingly, the 2 affected individuals from an Italian family presented with late-onset (age 47 and 57 years) asymmetric parkinsonism, early postural instability, and asymptomatic hepatomegaly,^3^ implying that the phenotypical spectrum of this disorder, with regard to both the neurological and hepatic manifestations, is wide. MRI of the brain typically shows hyperintensities in the basal ganglia and subthalamic and dentate nucleus on T1-weighted images, characteristic of manganese deposition.

The metabolic signature of this disorder is the extreme hypermanganesemia with polycythemia and depleted iron stores (e.g., low ferritin and increased total iron binding capacity), whereas laboratory findings reflecting hepatic dysfunction vary even between members of the same family.^1^, ^3^ Manganese induces erythropoietin gene expression and this could be the mechanism leading to polycythemia.^4^ The depleted iron stores can be explained by the fact that hypermanganesemia favors the release of iron from intracellular stores, enhances iron uptake, and decreases iron utilization.^5^–^8^

Here, we present longitudinal videos, clinical data, serial MRI images, and treatment response to chelation therapy over 10 years of the originally described case with a proven homozygous mutation in *SLC30A10*.^2^

## Case Presentation

This 22-year-old lady of Arabic origin was born to healthy first-cousin parents from a normal pregnancy and had an uncomplicated delivery and neonatal period. At the age of 2 years, she developed difficulty walking, which subsided for some years, but worsened again at the age of 11. At 12 years, she was mildly icteric with palpable hepatomegaly. Neurological examination revealed generalized dystonia, reduced arm-swing when walking, and increased tone in all four limbs, but no spasticity and no bradykinesia^2^ (see Video, Segment 1). She had one brother, who presented with a similar clinical syndrome and died after complications of cirrhosis at the age of 18, and seven healthy siblings.

**FIG. 1:**
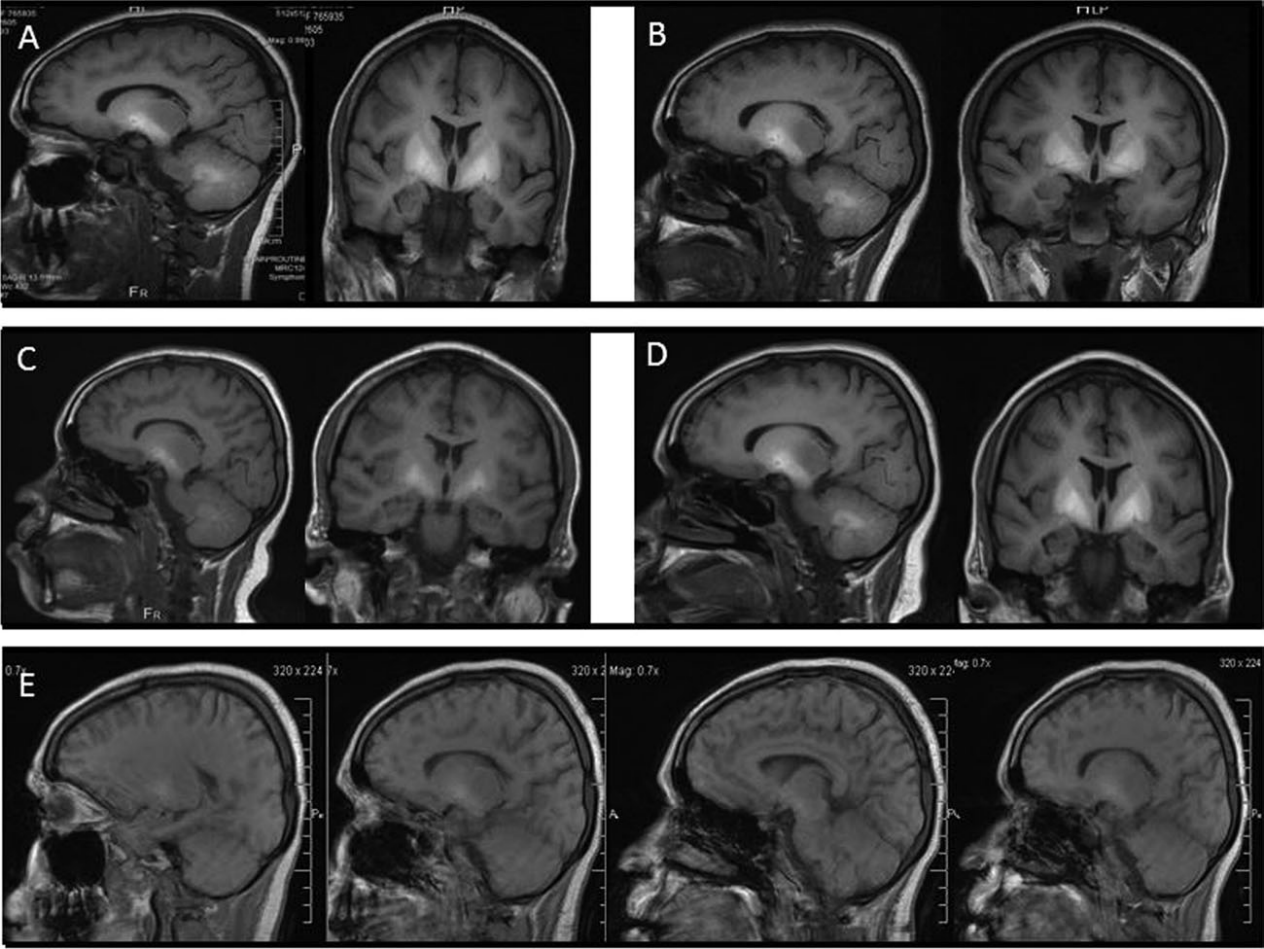
Serial MRI brain over a 10-year follow-up. (**A**) T1-sequences at the age of 12 (2002), before treatment. (**B**) T1-sequences at the age of 15 (2005), 3 years after treatment, slightly improved, compared to (**A**). (**C**) T1-sequences at the age of 16 (2006), 4 years under treatment, 1 year after increasing the frequency of infusions, and 1 month after adding oral ferrous iron, hyperintensities are less pronounced than before. (**D**) T1-sequences at the age of 20 (2010), 1 year after reducing chelation therapy, because of a lack of supplies. There is reaccumulation of manganese, compared to MRI 4 years earlier (**C**). (**E**) T1-sequences at the age of 21 (2011), 1 year after increasing the frequency of infusions, overall 9 years after onset of treatment, hyperintensities have clearly diminished.

Blood tests revealed polycythemia, hypermanganesemia, unconjugated hyperbilirubinemia, and increased total iron-binding capacity (TIBC) (Supporting Table 1).^2^ MRI of the brain (age 12; 2002) showed hyperintensities on T1-weighted sequences in the basal ganglia (caudate and lentiform nuclei), cerebellum (dentate nuclei and white matter), and anterior pituitary (Fig. 1A); hypointensities in these areas were present, to a much lesser extent, in T2-weighted sequences.^2^ Liver biopsy confirmed micronodular cirrhosis associated with elevated hepatic manganese content (Supporting Table 1).^2^ Recently, genetic testing has identified a homozygous mutation in the *SLC30A10* gene (nine-base deletion in exon 1 [c.314_c.322] resulting in a deletion of three amino acids [p.Ala105_Pro107] of the protein).^1^

Initial treatment with levodopa (120 mg/day) led to a mild improvement of dystonia. Vitamin E (100 mg/day) and a multivitamin preparation (Ketovite) were started to recover low vitamin E levels (Supporting Table 1). Chelation therapy with oral D-penicillamine (250 mg/6-hourly over 24 hours) led to a mild increase of urinary manganese excretion (from <91 nmol before treatment to 240 nmol after 24 hours).^2^ In contrast, intravenous disodium calcium edetate (CaNa_2_-EDTA [ethylenediaminetetraacetic acid]), 1 g twice-daily (BD) over 5 days led to significantly increased 24-hour urinary manganese (12,852 nmol after 5 days) and reduced blood manganese levels (from 2,800 to 1,780 nmol/L after 2 months) and improvement of dystonia.^2^ Hence, CaNa_2_-EDTA was the treatment of choice and continued as a 5-day monthly course. Zinc levels decreased during chelation treatment therefore zinc sulphate was added (125 mg BD).

At the age of 15, after 3 years of treatment, icterus had resolved and dystonia moderately improved (Supporting Table 1; see Video, Segment 2). However, brain MRI had only slightly improved (age 15; 2005) (Fig. 1B), and blood manganese levels were still high (2,322 nmol/L); therefore, CaNa_2_-EDTA was increased to 8 days/month. Oral ferrous fumarate (Fe) (204 mg/day) was added to decrease intestinal manganese absorption.^8^ One year after these adjustments (age 16; 2006), blood manganese had dramatically fallen; MRI (Fig. 1C) and liver histology had improved (Supporting Table 1). To avoid Fe toxicity, dosage was reduced because serum iron had reached abnormally high levels.

Because of a lack of supplies in her home country, she was treated less frequently with both CaNa_2_-EDTA and Fe over the duration of 1 year (from 19 to 20 years). This led to worsening of dystonia, increase in blood manganese levels, and more prominent hyperintensities in brain MRI (age 20; 2010) (Fig. 1D). Treatment was reintroduced and, at the age of 21, dystonia had improved (see Video, Segment 3). MRI showed reduced hyperintensities, compared to previous MRI images (age 21; 2011) (Fig. 1E). No susceptibility-related signal loss was evident on gradient echo images. Dopamine transporter imaging (DaTSCAN) was normal.

## Discussion and Conclusion

Here, we present the clinical description over 10 years, serial brain imaging, and response to chelation treatment of a patient with generalized dystonia, hypermanganesemia, cirrhosis, and polycythemia resulting from *SLC30A10* mutations.^2^ Diagnosis of this inherited metal storage disorder should not be missed because it is potentially treatable. Therefore, as for Wilson's disease (WD), we suggest that manganese blood levels should be routinely tested alongside copper and ceruloplasmin in the initial diagnostic work-up of patients with young-onset generalized dystonia.

Inherited disorders with mineralization evident on brain imaging may cause a variety of neurological syndromes, with different age of onset, phenotypes, and associated systemic abnormalities.^9^ These comprise WD, as a result of mutations in the *ATP7B* gene, encoding a copper transport ATPase,^10^ brain iron-accumulation syndromes (NBIAs),^11^, ^12^ syndromes with brain calcium depositions (e.g., Fahr's disease),^13^ and the syndrome described here with manganese accumulation. In contrast to manganese deposition, characterized by hyperintensities in T1-sequences, brain MRI in WD shows, among other features, hyperintensities in the basal ganglia in T2-sequences and the pathognomonic “giant panda face” in the midbrain^14^; NBIAs cause distinct patterns of iron deposition for each disorder in T2* sequences,^15^, ^16^ whereas calcifications are best evident as hyperintensities in CT. A phase II study with the iron chelator, deferiprone, in pantothenate kinase-associated neurodegeneration showed reduced iron in MRI after treatment, but no clinical improvement.^17^ Hence, WD and *SLC30A10* mutations are, to date, the only treatable conditions among these disorders.

Hypermanganesemia with brain manganese accumulation has been described in environmental overexposure (e.g., miners and smelters), in acquired hepatocerebral degeneration (AHD), after use of ephedrone, containing potassium permanganate, and in patients receiving parenteral nutrition.^18^–^22^ The clinical characteristics of these conditions may vary (Table 1).^18^, ^22^–^27^ For example, patients with environmental manganism present typically with parkinsonism, early postural instability, and psychiatric features, whereas in AHD, ataxia and orobucco-lingual dyskinesias are common. The distribution of hyperintensities in T1-MRI sequences are similar and cannot differentiate between these disorders.^28^, ^29^ Similarly, DaTSCAN is normal in both secondary hypermanganesemia and patients with *SLC30A10* mutations.

**Table 1:** Clinical characteristics, laboratory findings, and response to various treatments in manganism resulting from various etiologies

Cause of Hypermanganesemia With Brain Manganese Accumulation	*SLC30A10* Mutations^1^–^3^,^35^	Environmental Overexposure^20^,^23^,^27^,^28^,^32^–^34^,^37^,^38^	AHD^18^,^19^,^30^,^39^	Ephedrone^22^,^29^,^40^,^41^	Parenteral Nutrition^21^,^42^–^46^
Age at onset	Typically childhood (two adults)^3^	−	Typically >50 (rarely children)	−	−
Family history	Recessive	Typically negative	Typically negative	Typically negative	Typically negative
Clinical features					
Bradykinesia/rigidity (typically symmetric)	+/−	+++	+	+++	+
Postural instability (typically early)	+/−	+++	+	+++	+
Tremor (rarely rest-tremor; mostly postural-, action tremor)	+/−	+	+	+/−	+/−
Dystonia	+++	++	+	++	++
Chorea	−	−	+	−	−
Myoclonus (mainly asterixis)	−	−	++	+/−	−
Dyskinesias (typically orobucco-lingual)	−	−	+++	+	−
Slowing of vertical saccades—SGP	−	−	−	+	−
Dysarthria	+	+	+	+	+
Ataxia	−	−	+++	−	−
Spasticity	(+) One patient^35^	− (In some brisk reflexes)	− (In some brisk reflexes)	(+) One patient^40^	− (In some brisk reflexes)
Neuropsychiatric features	(+) One patient^47^	+	+++	++	+/−
Cognitive dysfunction	−	+/−	++	+/−	−
Laboratory findings					
Manganese serum levels (normal: <320 nmol/L)	↑↑↑ (e.g., range: 1,145−6,370)^1^	↑ (e.g., <2,000)	↑ (e.g., range: 379−989)^19^	↑ (e.g., active- [201−2,102; former-users <727]^30^)	↑ (e.g., range: 615–1,840)^45^
Depleted iron stores	+	−	−	−	−
Polycythemia	+	−	−	−	−
Liver dysfunction	+/−	−	+	−	+/−
Treatment					
Levodopa^a^(PO)	+/−	+/−	+/−	−	+/−
CaNa_2_-EDTA (IV)	++	+/−	Not tried	+/−	+
Dimercaptosuccinic acid (IV)	(+) Two siblings^1^	−	Not tried	Not tried	Not tried
Para-aminosalicylic acid (IV)	(−) One patient^47^	+/−	Not tried	Not tried	Not tried
Trientine (PO)	Not tried	Not tried	(+) One patient^36^	Not tried	Not tried
D-penicillamine (PO)	(−) One patient^2^	Not tried	Not tried	Not tried	Not tried
Other	+++Oral iron supplementation	−	Lowering blood ammonia, branched-chain amino acids, liver transplantation	Substance abstinence, amantadine, clonazepam, cerebrolysin^b^^48^	Supplement withdrawal

a +Mild to moderate effect; rarely formally tested, mostly in combination with other treatments.

b Cerebrolysin is produced by enzymatic breakdown of purified brain proteins and consists of low-molecular-weight peptides and amino acids.

Abbreviations: SGP, supranuclear gaze palsy; PO, per oral; IV, intravenously, AHD, acquired hepatocerebral degeneration.

However, there are certain laboratory findings underpinning the syndrome described here that should prompt testing for *SLC30A10* mutations in patients with hypermanganesemia. First, hypermanganesemia in *SLC30A10* mutations is usually much higher than in other causes of manganism (Table 1).^1^, ^3^, ^22^, ^26^ Second, polycythemia and depleted iron stores (e.g., low ferritin and high TIBC), as observed in patients with *SLC30A10* mutations, have not been observed in other causes of manganism (Table 1). Among the various causes of manganism, the main cause that may need to be differentiated from *SLC30A10* mutations is AHD, in particular, when the etiology of the primary liver dysfunction is unclear (e.g., cryptogenic liver cirrhosis), whereas the diagnosis of environmental overexposure, ephedrone use, and parenteral nutrition is facilitated by history, in most cases. These parameters may therefore be helpful to correctly identify patients with *SLC30A10* mutations. Moreover, because the full phenotypical spectrum of this disorder is, as yet, not known, these parameters may help in identifying patients with other phenotypical manifestations that may belong to this syndrome.

With regard to treatment, this should be initiated early and continued lifelong because the disorder may otherwise be fatal as a result of cirrhosis, but also to alleviate disability, because several patients have become wheelchair bound, when remaining untreated.^1^ CaNa_2_-EDTA infusions were effective in our and further patients with *SLC30A10* mutations,^1^, ^3^ whereas the effect may vary in patients with other causes of manganism (Table 1).^22^, ^25^, ^30^, ^31^

Dimercaptosuccinic acid, a further chelating agent, has been suggested to have some effect in 2 siblings with *SLC30A10* mutations,^1^ but not in manganese overexposure.^32^, ^33^ Sodium para-aminosalicylic acid may also act as a manganese-chelating agent and has been shown to be useful in patients with environmental manganese exposure and ephedrone users,^34^ but was not beneficial in 1 patient with *SLC30A10* mutations.^1^, ^35^ With regard to chelators commonly used in WD, D-penicillamine did not seem to be the treatment of choice in our patient, whereas 1 patient with manganism resulting from environmental overexposure has been reported to have improved with trientine (Table 1).^36^ Chelation therapy needs strict monitoring of other essential heavy metals, such as zinc, that may need to be supplemented. Moreover, additional oral iron supplementation seems to be crucial in the treatment of this syndrome because it limits intestinal dietary manganese absorption by competing with manganese for similar transport proteins (e.g., divalent metal transporter-1 and the transferrin/transferrin receptor system).^8^

Identification of further families and individuals with *SLC30A10* mutations will be important to unravel the true phenotypical spectrum and evolution of this disorder. Moreover, it would be of interest to investigate patients with manganism attributed to other causes for mutations or polymorphisms in this gene, which may explain why some individuals may be more prone to develop manganism than others when overexposed to manganese. Finally, further chelating agents, preferably administrable orally, and long-term follow up of various treatment outcomes are needed.

## Legends to the Video

**Video Segment 1.** The patient at the age of 12 before treatment, with dystonia affecting the limbs more on the right than the left, slowness of finger movements without true bradykinesia, dystonic gait, and reduced arm swing on both sides when walking.

**Video Segment 2.** The patient at 15, 3 years under treatment with mild improvement of dystonia, mostly on the right hand and improved gait.

**Video Segment 3.** The patient 9 years under treatment, with dystonic grimacing and dystonia of the limbs affecting more the left side. There is slowness in tapping, but no true bradykinesia. There is a slight terminal tremor on the left. There is in-turning of the right foot when walking.
