# Supplementary material for: Dystonia with Brain Manganese Accumulation Resulting From SLC30A10 Mutations: A New Treatable Disorder
Source: Mov Disord. 2012 Aug 23;27(10):1317–22. doi: 10.1002/mds.25138 (PMC3664426; doi:10.1002/mds.25138)
Supplement: Supplementary file 1 [file mds0027-1317-SD1.doc]

| **Age (years)**  **(Year)** | **12**  **(2002)** | **15**  **(2005)** | **16**  **(2006)** | **18**  **(2008)** | **20**  **(2010)** | **21**  **(2011)** |
| --- | --- | --- | --- | --- | --- | --- |
| **Treatment** |  | | | | | |
| **Supportive treatment** | Levodopa 30 mg qds  Vit E 100 mg od | Levodopa 50 mg tds  Vit E 100 mg od  Zinc 125 mg bd | Levodopa 50 mg tds  Vit E 100 mg od  Zinc 125 mg bd | Levodopa 50 mg tds  Vit E 100 mg od  Zinc 125 mg bd | Levodopa 50 mg tds  Vit E 100 mg od  Zinc 125 mg bd | Levodopa 50 mg tds  Vit E 100 mg od  Zinc 125 mg bd |
| **Iron supplementation** | - | - | Ferrous fumarate 68 mg tds  (for 1 month) | ↓ Ferrous fumarate 45 mg tds  (2006-2008) | Poor compliance from 2009 | Ferrous fumarate 45 mg tds  (2010-2011) |
| **Chelation therapy** | - | CaNa_2_-EDTA 1 g bd 5 days/month  (2002-2005) | ↑CaNa_2_-EDTA 1 g bd 8 days/month  (2005-2006) | CaNa_2_-EDTA 1 g bd 5-8 days/month  (2006-2008) | Poor compliance from 2009 | CaNa_2_-EDTA 1 g bd 5 days/month  (2010-2011) |
| **Investigations**  (normal range) |  | | | | | |
| **Whole blood manganese**  (<320 nmol/L) | ***3285*** | ***2322*** | 171 | ***578*** | ***2844*** | Not done |
| **Hemoglobin**  (11.5-15.5 g/dL) | ***16*** | 14.7 | 13.7 | 12.7 | ***17.8*** | 13.4 |
| **Serum iron**  (5-25 µmol/L) | 22.1 | 18.7 | ***50.9*** | Not done | ***60.1*** | ***29.8*** |
| **Total iron binding capacity**  (50-90 µmol/L) | ***>120*** | 87.4 | 69 | Not done | ***114.2*** | 44 |
| **Ferritin**  (7-150 µg/L) | ***8*** | ***8*** | Not done | 46 | 21 | ***165*** |
| **Prothrombin time**  (9.6-11.8 sec) | ***12.9*** | ***11.9*** | Not done | 10.7 | ***12.1*** | ***12.7*** |
| **aPTT**  (26-38 sec) | ***41.3*** | 37.9 | Not done | 35.9 | 37.4 | 32.1 |
| **Unconjugated bilirubin**  (<18 µmol/L) | ***45*** | ***49*** | ***25*** | ***24*** | ***55*** | ***29*** |
| **ALP**  (40-120 U/L) | ***371*** | ***144*** | 89 | 67 | 120 | 52 |
| **Vitamin E**  (11.5-35 µmol/L) | ***11.3*** | Not done | Not done | 18.2 | 27.2 | Not done |
| **Plasma Zinc** (11-18 µmol/L) | ***10.2*** | 18.8 | Not done | 12.4 | 11.3 | Not done |
| **Hepatic manganese**  (1-2 µg/g wet weight) | ***3.4*** | Not done | 1 | Not done | Not done | Not done |
| **MRI Brain: Figure** | 1A | 1B | 1C | Not done | 1D | 1E |

**Supplementary Table:** **Treatment and laboratory findings over a 10-year follow-up of the patient with *SLC30A10* mutations.**

Abbreviations: Od, once a day; bd, twice a day; tds, three times a day; qds, four times a day; Vit E, Vitamine E; CaNa_2_-EDTA, disodium calcium edetate infusions; ALP. Alkaline phosphatase; MRI, magnetic resonance imaging
